# Use of author identifier services (ORCID, ResearcherID) and academic social networks (Academia.edu, ResearchGate) by the researchers of the University of Caen Normandy (France): A case study

**DOI:** 10.1371/journal.pone.0238583

**Published:** 2020-09-02

**Authors:** Christophe Boudry, Manuel Durand-Barthez

**Affiliations:** 1 Normandie Univ, UNICAEN, Média Normandie, Caen, France; 2 Unité régionale de formation à l’information scientifique et technique (URFIST), Ecole Nationale des Chartes, PSL Research University, Paris, France; 3 Laboratoire “Dispositifs d’Information et de Communication à l’Ère Numérique” (DICEN), EA7339, Conservatoire National des Arts et Métiers, Paris, France; University of Sydney, AUSTRALIA

## Abstract

The purpose of this paper was to assess the presence of researchers on two author identifier services (ORCID and ResearcherID) and to compare the results with two academic social networks (Academia.edu and ResearchGate) using the categories of discipline, career advancement, and gender in a medium sized multidisciplinary university in France (University of Caen Normandy). Metrics such as number of publications per researcher, h-indexes, and average number of citations were also assessed. Of the 1,047 researchers studied, 673 (64.3%) had at least one profile on the four sites, and the number of researchers having multiple profiles decreased as more sites were studied. Researchers with only one profile numbered 385 (36.8%), while 204 (19.5%) had two, 68 (6.5%) had three, and only 16 (1.5%) had four. ResearchGate had by far the highest number of researchers present, with 569 (54.3%), whereas presence on the other sites was about 15%. We found that, apart from Academia.edu, researchers in Sciences, Technology, and Medicine (STM) were over-represented. Overall, experienced male researchers were over-represented on the sites studied. Our results show that, because of the numerous profiles lacking publication references (particularly on ORCID) and a low presence of researchers on the four sites studied (except for ResearchGate), assessing the number of publications, h-indexes, or average number of citations per article of individuals or institutions remains challenging. Finally, our data showed that French researchers have not adopted the use of the two author identifier sites (i.e. ORCID and ResearcherID). As long as French researchers remain reticent, these sites will not be able to provide the services for which they were created: addressing the problem of author misidentification, consequently providing exhaustive access to scientific production and bibliometric indicators of individual researchers and their institutions.

## Introduction

To assess researchers’ scientific output, several online bibliographic databases have been available (since the late 1990s), but access to nearly all of them is limited by a paywall (both the Web of Science and Scopus are expensive registries, limiting access to a relative minority). The sites are compartmentalized, often thematic, and incomplete by definition. Some repositories (geographically limited and often unknown to researchers) offered, in the early 2000s, free access to full texts with open access. Nevertheless, these tools of the era lacked a fundamental feature: direct communication between those actively doing research and other researchers. These sites required intermediate operations, such as obtaining the authors’ email by searching for articles in which they were declared as the corresponding author or searching on the internet on what appeared to be an author’s personal/institutional page, then having to send an email to contact them. Another drawback of these bibliographic databases was that their content could not be controlled and corrected by adding references to researchers if necessary. In 2008, two Academic Social Networks (ASNs), ResearchGate and Academia.edu, appeared almost simultaneously, responding favorably to these expectations. They allowed researchers to communicate directly with their peers with an internal and “proprietary” mailing system, and adding their scientific publications onto their profiles (often in full text, at the risk of infringing publishers' contracts) [1,2]. These interfaces also offer several benefits often ignored by conventional tools:

- Significantly increasing visibility rate by sharing publications (references and full texts) and information, thus contributing to the building of their reputation [3,4]
- Automatically alerting users to the addition of new publications considered to be of interest
- Allowing connection and collaboration with colleagues and experts in the field
- Asking and answering questions and even finding suitable job opportunities
- Serving as a source of bibliometric as well as altmetric indicators such as publication counts, reads, number of downloads, citations, and profile views

ASNs such as Academia.edu or ResearchGate do not, however, offer tools to recover researchers in an unambiguous manner. Free but “watertight”, these sites have the common feature of an extremely basic documentary operation. That is, the search key is the author’s name, which obviously conditions the display of the least approximate answer possible. However, this key is limited to raw character strings on these networks, leaving the door open to excessive atomization of identities, often limited due to a lack of relevant responses despite a lexical interpretation based on conventional character matches.

The accurate identification of researchers and their scientific production is, however, crucial for all parties involved in research (e.g. publishers, funders, universities, research evaluators, libraries) because many actions depend on the precision of this step (e.g. promotions, obtaining funds, publishing or reviewing articles). Nevertheless, recovering all articles by a specific author or institution through the current proliferation of online journal articles sometimes feels like searching for a needle in the haystack [5]. Indeed, difficulties encountered in tracking scholarly and institutional publications are numerous due to identical or similar names, name changes over time due to marriage, or the use of aliases or author groups [6]. Change of researcher affiliations over time, due to researcher mobility and/or lack of uniformity when declaring affiliations in articles are also well known difficulties [7,8]. Different spellings of names can occur due to alternative transliterations of some author names from other alphabets (e.g. Cyrillic, Chinese, etc.) though some platforms such as Google Scholar allow authors to include different forms of their names, or SciFinder, from the Chemical Abstracts Service, allows a search for authors using alternate names [9].

For years, unique identifiers have been developed for concepts (e.g. concept codes of patent databases, especially the Cooperative Patent Classification and the Classification Tool of the Inspec bibliographic database from the Institution of Engineering and Technology, UK), material items (e.g. the Registry Number of Chemical Abstracts cover all written forms of chemical elements or compounds), books (e.g. International Serial Book Number), periodical journals (e.g. International Standard Serial Numbers), or articles (e.g. Digital Object Identifiers). Unique identifiers have also been developed for authors: in libraries, such as the International Standard Name Identifier (ISNI) or the Library of Congress Name Authority File (LC/NAF) in the USA, providing authorized name access points for monograph authors in library bibliographic records rather than journal article authors for economic reasons [10]. Relatively recently, as author identifiers are also essential to identify the scientific output of scholars, publishers, and organizations have developed Author identifiers (AIDs) for scholars. Scholarly repositories such as Research Papers in Economics (RePEc) in 1999 and arXiv in 2005 first included AIDs [11]. Then, Scopus Author Identifier (ScopusID) in 2006 and Web of Science ResearcherID in 2008 were developed by bibliographic database providers (Elsevier and Thomson Reuters, respectively). These AIDs were created to assign a unique researcher identification to bibliographic records in these databases. One must note that Open Access and new technologies such as Altmetrics were also highly involved in the emergence of these tools. For each of these AIDs, authors can check the automatically generated list of their publications, through which they can track their publications. Using the Scopus Author Identifier, only publications indexed in the database are present in the list of publications, making it impossible to generate a complete list. To overcome this problem and offer an AID independent of scholarly repositories and bibliographic databases, Open Researcher & Contributor ID (ORCID) was launched in 2012. ORCID is an open, international, non-profit, cross-national, community-based project that is supported by its membership fees [12]. ORCID allows researchers to enter any publications they wish into their profile and to control what data is entered. Unfortunately, one must note that the databases associated with Author Identifiers, despite the often precise indication of the authors’ affiliations, do not allow direct and immediate communication between researchers. Nevertheless, in addition to the advantage of assigning a unique researcher identifier to bibliographic records, the expected benefits of using AIDs, particularly those of ORCID, are numerous. They include: offering a hub for international services offered to researchers [13], reliably and easily connecting researchers: with their contributions and affiliations [14], and tracking scientific output [15,16].

Numerous articles have been published on ASNs, especially Academia.edu and ResearchGate [17], or to explain the usefulness of AIDs (e.g. [8,15,13,16]), but few studies have been conducted specifically to assess how AIDs are used by researchers [7,18,19]. It is important to examine the usage of these systems to know who and where the users of AIDs are, and to assess whether these tools could improve the characterization of institutional or researchers’ scientific output. The responses to these questions could be helpful in improving training and services. Moreover, they could help decision makers determine whether or not to promote the use of these tools.

The objective of the present study was to assess the presence of researchers on two AIDs (ORCID and ResearcherID) and to compare the results with two ASNs (Academia.edu and ResearchGate), categorized by discipline, career advancement, and gender in a medium sized multidisciplinary university in France (University of Caen Normandy). The simultaneous presence of researchers on these four sites was evaluated to assess the ability of researchers to maintain multiple profiles. The total number of publications found on each of the four sites studied was assessed and compared to the number of publications found in the Scopus database. This was done to evaluate the ability of the sites to provide an accurate view of the scientific output of individuals and their institution. The total number of publications per researcher, h-indexes, and average number of citations per researcher were assessed on ResearchGate and ResearcherID by comparing them to the Scopus database to report the ability of these two sites to provide relevant metrics on individuals and their institution.

## Methods

### Service providers included in this study

The decision to include ORCID and ResearcherID in the present study resulted from a literature review and preliminary searches in several name identifier databases. We did not include discipline-dependent AIDs (e.g. ArXivID or RepecID) because our target population of researchers was multidisciplinary.

#### ORCID

Launched in 2012, ORCID is an open, international, non-profit, community-based project. It uses an open source and cross-national approach to author identification, and has more than 600 members and hosts more than 7.5 million active profiles. Researchers must register to use ORCID and have to create their own profile. They can further manually add information such as affiliation, employment, funding, publications, and peer reviews. Contrary to other AIDs, ORCID is interoperable with numerous organizations, allowing automatic updating of information from other sites (e.g. CrossRef or Scopus for publications or Publons for peer reviews). To increase interoperability with other AIDs, researchers can add a link to external identifiers in their profiles, found under “Other IDs”. As the creation of profiles is not supervised or controlled, researchers can create multiple profiles, leading to duplication. Furthermore, some authors have pointed out ORCID’s vulnerability to fraud and hacking [20]. ORCID is promoted by most publishers (such as PLOS and Wiley) and journals [21], and it is required for submitting articles to most submission platforms (e.g. Scholar One [13,22], which is linked to about 5,000 journals in the world [16]). ORCID is also required by some national or international agencies for grant-funding requests. Finally, ORCID does not provide metrics and does not allow researchers to upload full texts of their publications.

#### ResearcherID

ResearcherID, now hosted by Publons.com, is offered by Clarivate Analytics’ Web of Science database and was introduced in 2008. To use ResearcherID, authors have to register and complete their profile. Publications from the Web of Science database are automatically added to the profile, but authors can also add publications from other databases, from DOIs, or by uploading files using Bibtex or RIS formats. Uploading full texts is not possible on ResearcherID. Researchers can add information about their affiliations, add keywords to describe their research field, add reviews, view their citations (based on publications on Web of Science), and view metrics. ResearcherID is interoperable with ORCID, automatically exchanging information (e.g. publications). Logging in ResearcherID is possible using ORCID log-in information. Researchers can also add a link in their profiles to external identifiers, found under “identifiers”.

#### ResearchGate

ResearchGate, which was launched in 2008, is an ASN that is mostly oriented towards the STM (Science, Technology and Medicine) fields [17,23,24]. SSH (Social Sciences, Arts & Humanities) are nevertheless present as a minority. ResearchGate offers complete services at no cost. There is no method for payment, unlike Academia.edu. This site offers specific metrics, such as the RG Score, the relevance of which is still under scrutiny [25,26]. It is based on the volume of a researcher’s’ publications as well as the amount of participation in exchanges they have with other researchers. We should add that ResearchGate provides a specific field that allows authors to specify an ORCID in their profile, found under the "Info" tab. It is visible, yet unusable as a search key. The main characteristics of ResearchGate are common to Academia.edu, and we provide a summary of them below.

#### Academia.edu

Launched in 2008, Academia.edu is an ASN mostly oriented towards Social Sciences, Arts & Humanities [17,27]. Academia.edu offers all the standard services provided by ASNs, but some information is available only in its paid version (e.g. direct and complete access to citations). Mention of an e-mail in the profile is not mandatory, and an ORCID may be mentioned in the CV (optional as well) without any dedicated field. Its numerical chain will not be taken into account as a research key. Nevertheless, Academia.edu suggests including alternative written forms of authors’ names.

We can now summarize some characteristics common to both Academia.edu and ResearchGate:

- Free and immediate deposit of references and full text publications without regard to aspects relating to authors' rights and publishers' contracts
- Use of an internal mailing system allowing direct contact
- Lack of interoperability between the two networks and other services (including AIDs) used by researchers

Table 1 provides a summary of the main characteristics of ORCID, ResearcherID, ResearchGate, and Academia.edu.

**Table 1:** Main characteristics of ORCID, ResearcherID, ReserchGate, and Academia.edu.

	ORCID	ResearcherID	ResearchGate	Academia.edu
**Accessibility**	Non-profit	For-profit company	For-profit company	For-profit company
	Access without restriction	To get an identification number, a minimum of one publication in Web of Science is mandatory	Access without restriction	Access without restriction
	Free of charge	Free of charge	Free of charge	Free of charge with paid version offering more features
**Institutional use and career links**	In practice, mandatory in most institutional approaches	Highly recommended in most institutional approaches besides ORCID	No	No
			Recommendations, job opportunities	Grants, job opportunities
**Interoperability with author identifiers**	Link/exchange of data with ResearcherID and Scopus ID	Link/exchange of data with ORCID	ResearcherID and ORCID optionally added by the researcher in the CV but are not search keys	ResearcherID and ORCID optionally added by the researcher in the CV but are not search keys
**Interconnection /researcher**	No	No	Internal mailing	Internal mailing
			Followers/Following	Followers/Following
**Researcher CV**	Yes	Yes	Yes	Yes
**Publications**	Diverse sources admitted (Crossref, ResearcherID, Scopus…) including personal. No full text upload / share by user.	ResearcherID links researcher’s publications across all Web of Science Group products. Addition of external items admitted, including personal. No full text upload/share by user.	Full text upload / share by user, if applicable. Copyright infringement recorded in the past	Full text upload/share by user, if applicable. Copyright infringement recorded in the past
**Citations**	No	Web of Science Core Collection Citations	Yes	Yes in paid version
**Analytics**	No views and/or download stats	No view and/or download stats	Views (publications), proprietary metrics (e.g. RG Score, Research Interest)	Views (publications and profile) and downloads (full texts) up to 6 months (more on paid version)

### Study setting and data collection

Situated in Normandy, approximately 250 kilometers west of Paris, the University of Caen Normandy offers programs in all academic disciplines to approximately 30,000 students. Forty-five laboratories are affiliated with the university: 28 Sciences, Technology, and Medicine (STM) and 17 Humanities and Social Sciences (HSS). Altogether, there were 1,047 researchers included in the study: 619 (59.1%) in STM and 428 (40.9%) in HSS. The number of researchers and laboratories per discipline, according to the Frascati Manual Field of Science and Technology classification/OECD [28], is presented in Table 2.

**Table 2:** Number of researchers and laboratories per search field at the University of Caen Normandy.

	STM/HSS	Number of laboratories	Number of researchers (%)	Number of researchers (%)
**Natural Sciences**	STM	10	191 (18.2)	619 (59.1)
**Engineering and Technology**	STM	6	238 (22.7)	
**Agricultural Sciences**	STM	1	15 (1.4)	
**Medical and Health Sciences**	STM	11	175 (16.7)	
**Social Sciences**	HSS	10	244 (23.3)	428 (40.9)
**Humanities**	HSS	7	184 (17.6)	
**Total**		45	1047	1,047

Name, surname, status, and laboratory of the 1,047 researchers were provided by University of Caen Normandy. Assistant professors, lecturers, and assistant researchers were considered as early career researchers, whereas full professors and research directors were considered as experienced researchers. For 48 individuals, the status was “other researchers” and did not allow us to determine career advancement. The discipline of each researcher was assigned according to the search field of their own laboratory. Gender was not provided by the University of Caen Normandy, but was determined by searching the internet.

We manually searched for each of the 1,047 researchers whether or not they had a profile on any site studied. Bibliometric indicators were collected as follows. The number of publications (references) on each profile were collected for the four sites studied. The h-index [29] and the number of citations per article were collected from ResearcherID and ResearchGate (not available on ORCID and Academia.edu). The number of publications, h-indexes, and number of citations per article were collected from the Scopus database for comparison purposes [24,30].

All searches were done in March 2019. As described previously by Sandberg et al. [10], ORCID searches were complicated when the name of the researcher was found in the ORCID registry but the profile was private, with no distinguishing information available. Because it was impossible to disambiguate or confirm researcher identity in these cases, as done previously [19], we did not include ORCID records that were not public, or when no information was available in the profile. On the Academia.edu site, each profile was checked to determine whether the profile was created by the researcher or done automatically by the site. Only profiles created by the researchers were included in the analysis. Spearman’s rank correlation coefficient (Spearman’s rho) was calculated using Microsoft Excel 2010 (Microsoft, Redmont, USA).

## Results and discussion

### Number of profiles and overlap between sites

The total number of ORCID records (private and public) found was 405 (38.7%), including 179 (17.1%) from public records, which were taken into account as explained in Methods.

Of the 1,047 researchers studied, 374 (35.7%) had no profiles on any of the four sites studied. The percentages of researchers who did not have any profiles on the two AIDs or ASNs were 75.1% (n = 786) and 40.2% (n = 421), respectively. These results showed that, even today, a large number of researchers are not interested in AIDs, and to a lesser extent, in ASNs.

The number of profiles and overlap between the different sites is presented in Table 3.

**Table 3:** Number of profiles and overlap between sites.

	ORCID	ResearcherID	ResearchGate	Academia.edu
**ORCID**	**24 (13.4)**	2 (1.1)	73 (40.3)	4 (2.2)
**ResearcherID**	2 (1.1)	**21 (13.8)**	53 (34.9)	0 (0)
**ResearchGate**	73 (40.3)	53 (34.9)	**287 (50.4)**	72 (12.7)
**Academia.edu**	4 (2.2)	0 (0)	72 (12.7)	**53 (32.9)**
**Total number of profiles (%)**	179 (17.1)	152 (14.5)	569 (54.3)	161 (15.4)

Columns 2 to 5: number of researchers having only the 2 profiles in the column and the row (e.g. 72 researchers have a profile on Academia.edu and on RG and do not have ORCID and ResearcherID profiles). Bold numbers on the diagonal: number of researchers having only one profile on each site. In parentheses: respective percentage relative to all profiles found on the site (e.g. 53 researchers have a profile only on Academia.edu. These are 32.9% of all the profiles we found on this site). Last row: total number. In parentheses: percentage of profiles on each site.

The number of profiles on the two AIDs were quite similar: from 152 (14.5%) for ResearcherID to 179 (17.1%) for ORCID. Surprisingly, while ORCID is considered as the “leading global scholarly ID registry” [31] and is “the de facto standard unique researcher identifier” [32], few researchers in the Caen University use this service (n = 179; 17.1%). This may be because, unlike other countries (e.g. Portugal) [33], France does not yet require or encourage researchers to register on ORCID (though France is expected to join ORCID soon [34]). As previously mentioned by Aman [35], our data show that, although ResearcherID has been available for almost a decade, most scientists have not taken advantage of the ResearcherID capabilities or even set up a profile. As shown in Table 4, our data are in accordance with previous studies on AIDs (apart from the Canadian study with 62% using ORCID; the sample of researchers was not randomly selected [19]), and confirm that ORCID and ResearcherID are little used by researchers today. As expected, among ASNs [17], the highest number of profiles by far was found on ResearchGate (n = 569; 54.3%). The number of profiles on Academia.edu was similar to that of the two AIDs.

**Table 4:** Percentage of researchers having a profile on Academia.edu, ResearchGate, ORCID, and ResearcherID. Comparison with former studies.

	Country	Number of persons included in the study	ORCID	ResearcherID	ResearchGate	Academia.edu
^1^**Haustein et al. (2015)** [36]	International*	57	35	N/A	58	30
**Mikki et al. (2015)** [18]	Norway*	4307	3	3	30	4
^2^**Sandberg (2016)** [10]	International*	291	14.4	N/A	N/A	N/A
**Tran (2017) n = 335** [7]	USA***	335	15	7	31.5	12
^3^**Aman 2018** [35]	International*	193	20.7	24.9	N/A	N/A
**Morgan 2018** [19]	USA	50	12	N/A	N/A	N/A
			62			
	Canada**					
**Our study 2019**	France**	1047	17.1	14.5	54.3	15.4

Methods:

*automatic search on databases.

**manual search on databases

*** Survey.

^1^Persons included in this study were bibliometricians who are probably more aware of these tools than other researchers, which probably explains why the presence of these researchers is higher than in other studies.

^2^Authors of articles in 3 journals (Cataloguing & Classification Quarterly, Perspectives of New Music and IEEE Intelligent Systems).

^3^laureates of Leibniz Prize between 1999 and 2016.

As expected, the number of researchers having multiple profiles decreased when the number of sites increased: 385 (36.8%) researchers only had one profile, 204 (19.5%) had two profiles, 68 (6.5%) had three profiles, and only 16 (1.5%) had four. As previously mentioned [18], this is due to the difficulty of updating several profiles. Consequently, most researchers only managed one profile [23,37]. This could be easily improved with better interoperability between these sites, but we must note that, apart from ORCID, and to a lesser degree ResearcherID, none of the sites are moving in this direction.

### Number of profiles by scientific disciplines

The number of researchers who had no profile on the four sites studied was 374 (35.7%). More than half of the researchers in HSS (n = 221; 51.6%) and less than a quarter of the researchers in STM (n = 153; 24.7%) had no profiles on any of the four sites studied, clearly showing that HSS researchers are less active on AID and ASN sites.

**Fig 1:**
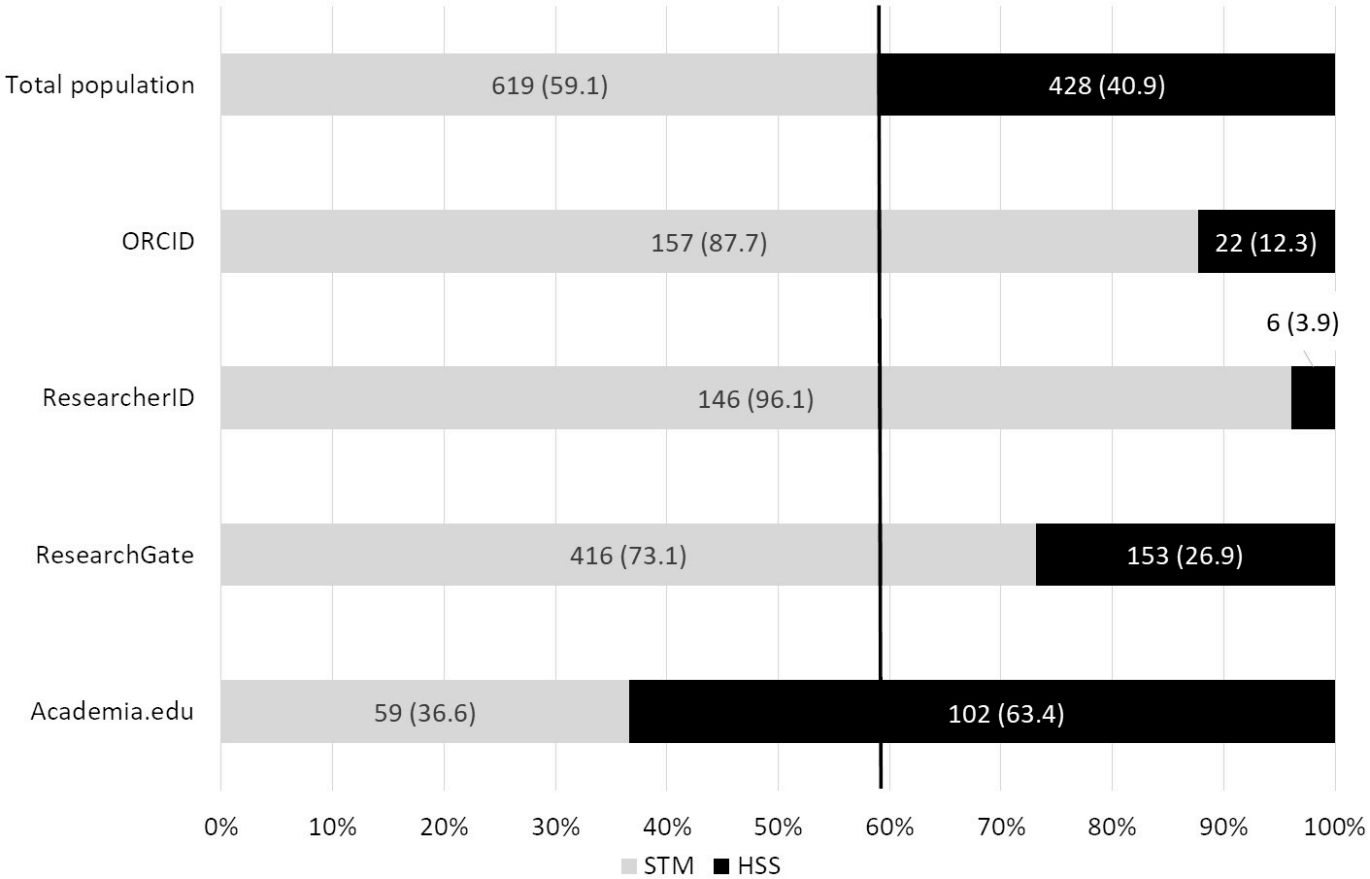
Number of profiles by discipline on the four sites studied. Percentages are shown in parentheses. Vertical line: Reference value for the whole population indicating the over- or under-representation of STM researchers vs total population. STM researchers are under-represented on Academia.edu, and over-represented on ResearchGate, ORCID, and ResearcherID.

The presence of researchers on the four sites grouped by scientific disciplines (i.e. STM or HSS) is presented in Fig 1. As previously described, our data show that HSS researchers are over-represented on Academia.edu [17,27], whereas STM researchers were over-represented on ResearchGate [7,17,18,23,38]. However, regardless of the fact that ORCID and ResearcherID are not known to be specifically dedicated to HSS or STM, STM researchers were over-represented on them. The reason for the use of ORCID may be that several services, such as journal submission platforms, mainly used by STM researchers, require ORCID registration.

**Fig 2:**
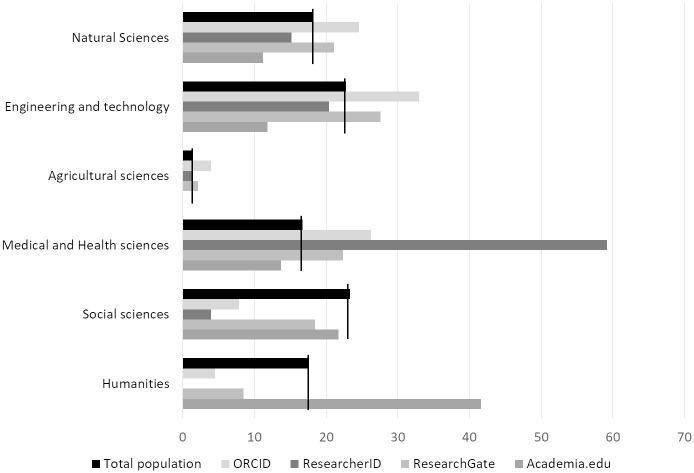
Percentage of profiles on the four sites studied grouped by discipline.

When considering the presence of researchers on the four sites studied grouped by discipline, we notice that, apart from researchers in Humanities, all disciplines were under-represented on Academia.edu, even researchers in Social Sciences (Fig 2).

This suggests, as previously described by Mikki et al. [18], that there are differences in behavior among researchers in Social Sciences and Humanities using Academia.edu which are hidden when considering HSS as a whole. Results for ResearchGate are in accordance with previous studies [23,27], showing the predominance of biologists on this site. All STM disciplines were over-represented on ORCID, unlike HSS. Results for STM disciplines, apart from Medical and Health Sciences, were less significant for ResearcherID.

### Number of profiles relating to career advancement

**Fig 3:**
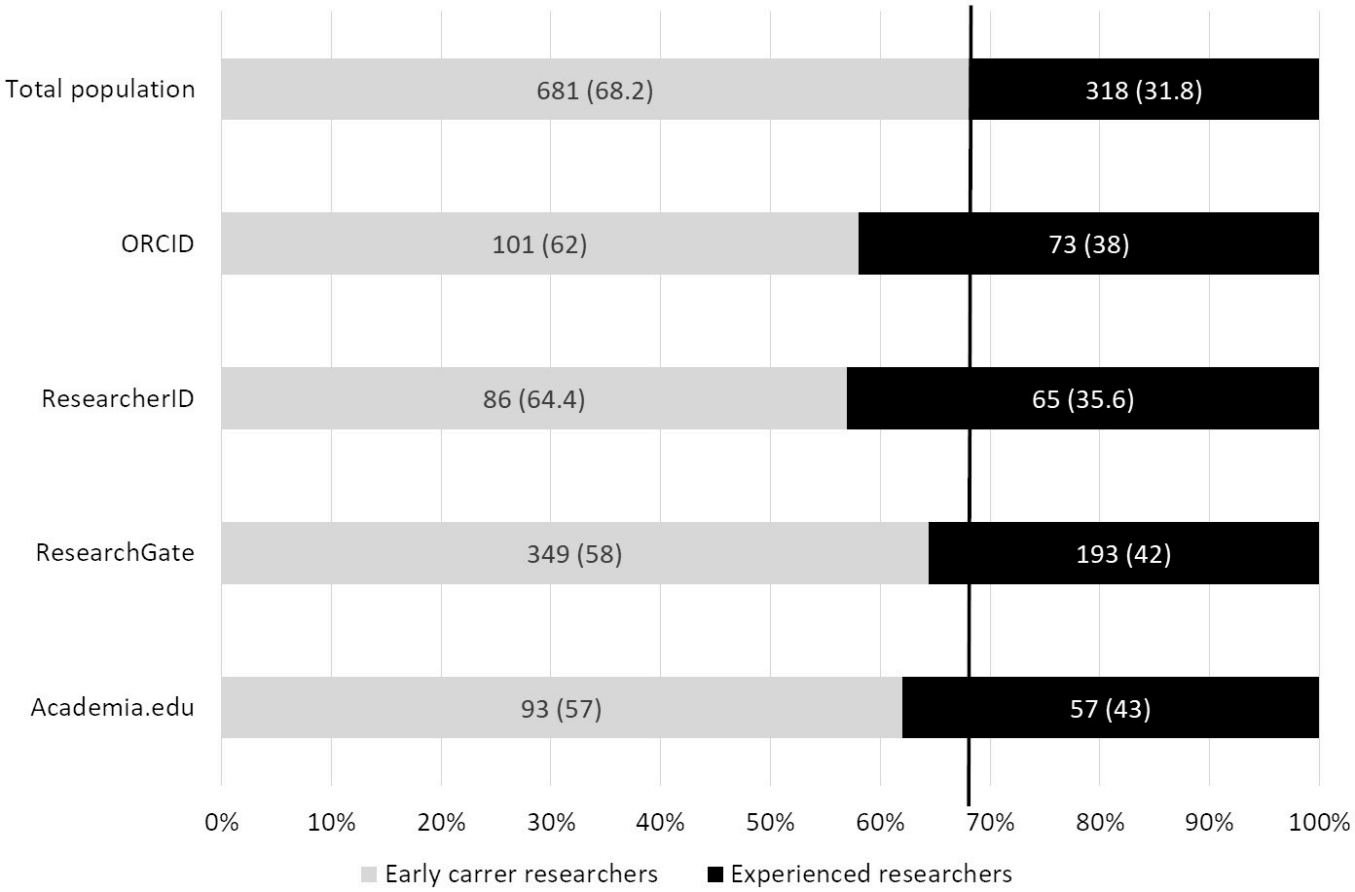
Number of profiles on the four sites categorized by career advancement. Vertical line: Reference value for the whole population. Percentages are shown in parentheses.

Of the 1,047 researchers included in this study, career advancement for 48 individuals could not be determined. Of the 999 researchers for whom career advancement was found, 681 were in the early stages of their career and 318 were experienced researchers. The percentage of early career researchers who had no profiles on any of the four sites was 44% (n = 300), compared to only 26.1% (n = 83) for experienced researchers. Furthermore, experienced researchers were over-represented on the four sites, particularly on ResearcherID (Fig 3), suggesting that they are more interested in creating profiles on AIDs and ASNs. These results have already been demonstrated in Mikki et al. [18], in which professors had the highest presence on ASNs and AIDs. This is surprising since early career researchers are among those theoretically most in need of visibility to promote and build their reputation [4]. Although their presence on AIDs and ASNs would be highly advantageous, they are the least likely to use these services.

We must note that our study did not include postdoctoral researchers or PhDs as early career researchers, who were mentioned as being “not convinced of the value of the social media” in France [39]. Including these two categories in “early career researchers” would certainly have modified the results obtained.

### Number of profiles related to gender

**Fig 4:**
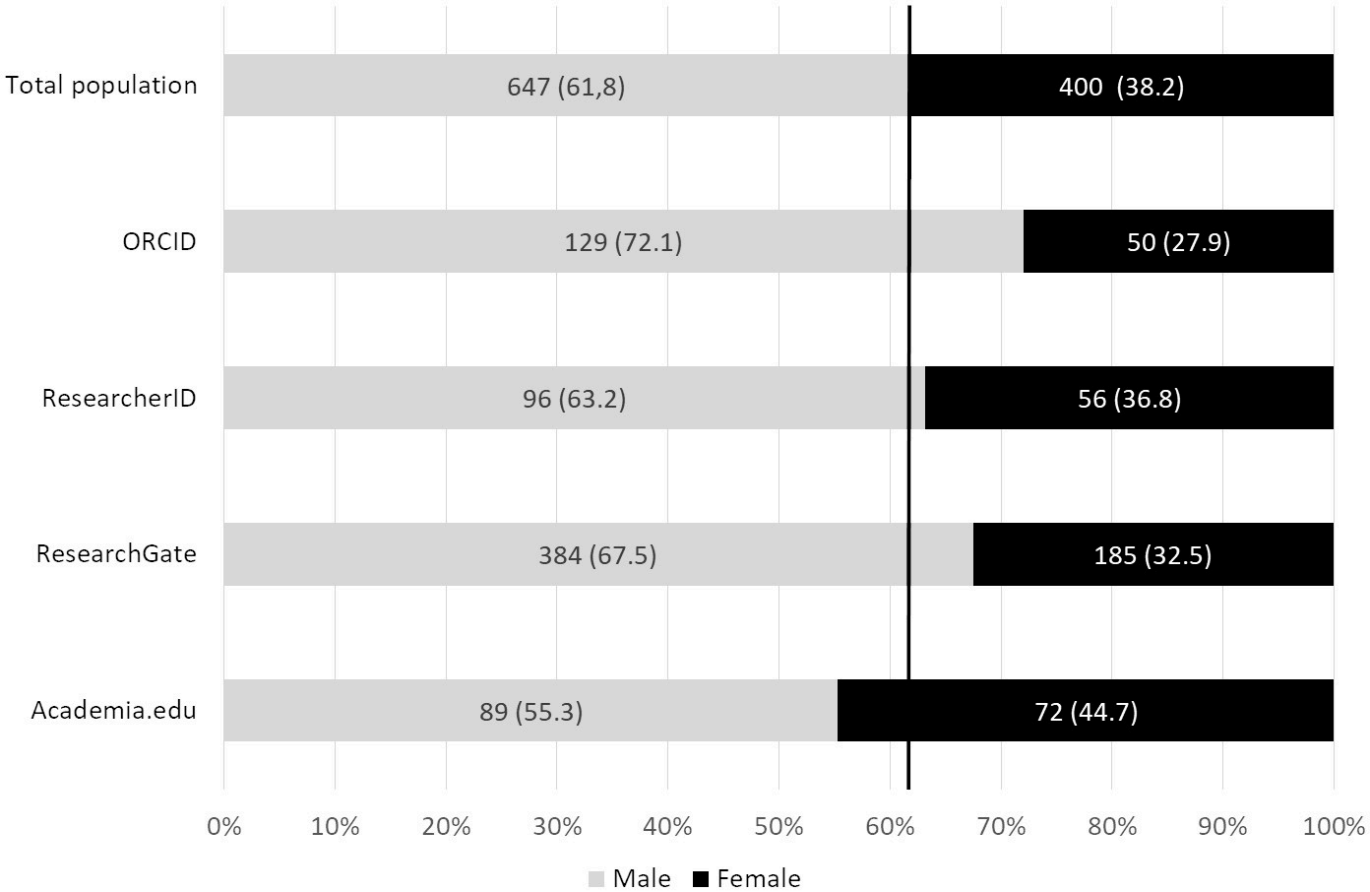
Number of profiles on the four sites relating to gender. Vertical line: Reference value for the whole population. Percentages are shown in parentheses.

Of the 1,047 researchers included in this study, there were 647 men and 400 women. 41.8% (n = 167) of the women and 32% (n = 207) of the men had no profiles on any of the four sites studied. Thus, there was an under-representation of women on ASNs and AIDs, which is in line with some earlier findings [18]. However, we note that, according to findings from other studies, it is not clear whether there are similar gender differences in academic social website use. [27]. As shown in Fig 4, women were nevertheless over-represented on Academia.edu. These data do not seem to show that women have a preference for creating a profile on Academia.edu. It is rather due to the indirect effect of the higher proportion of women in HSS and the fact that HSS researchers were over-represented on Academia.edu (the percentage of women in HSS was 51.9%, whereas the percentage of women in STM was 28.8).

### Number of publications

Overall, the total number of publications found in the profiles of the Caen University researchers amounted to 5,303 on ORCID, 7,815 on ResearcherID, 37,100 on ResearchGate and 6,349 on Academia.edu compared to 35,593 in the Scopus database. This means that using Academia.edu, ORCID, and ResearcherID to assess the total number of publications produced by researchers from Caen University would lead to a large underestimation of research output from this institution.

**Fig 5:**
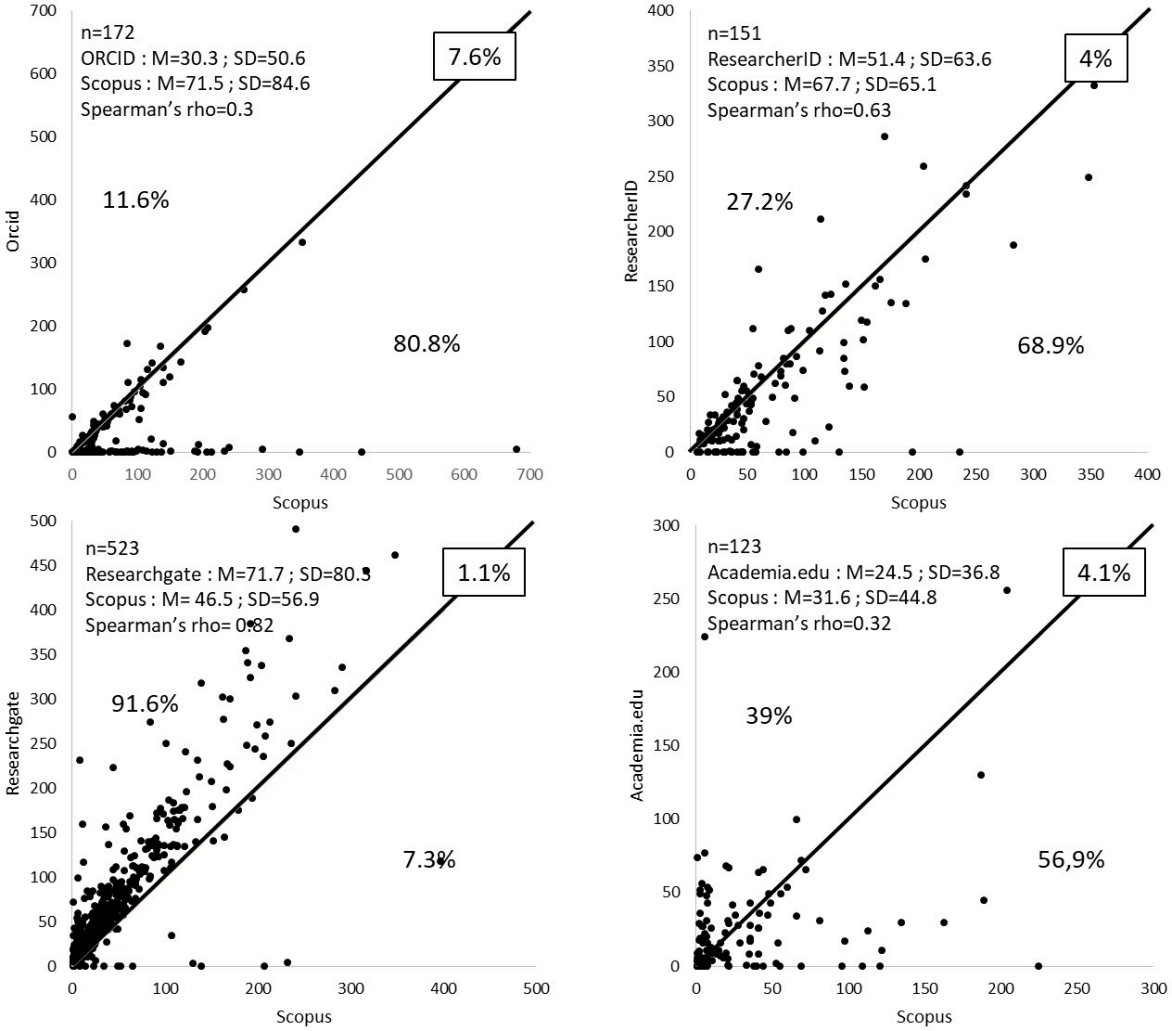
Number of publications referenced by researchers on the four sites compared to the number on Scopus. M and SD: Mean and standard deviation. The numbers above, on, and below the line indicate the percentage of researchers having more, the same, or fewer publications referenced on the studied sites compared to Scopus, respectively.

The number of publications per individual found for researchers on the four sites compared to the Scopus database is presented in Fig 5. For the two AIDs, ORCID and ResearcherID, the average number of publications per researcher is generally lower than that found on Scopus (30.3 instead of 71.5 for ORCID and 51.4 instead of 67.7 for ResearcherID). Please note that the value obtained with ResearcherID is closer to the reference value on Scopus because the profiles on ResearcherID benefit from the automatic addition of the publications present in the Web of Science database. Furthermore, using these sites to assess the number of publications by researchers leads to an underestimation of the number of publications for 80.8% and 68.9% of the researchers (Fig 5). We found a large number of profiles without any publications on ORCID: 58 profiles of 179 (32.4%). This has already been described by Sandberg et al. [10], Youtie et al. [33] and Morgan et al. [40] for 79.4% of profiles in 2016, for 67.9% in 2017, and 44.8% in 2018, respectively. This percentage of profiles without publications seems to be decreasing with time, but is still substantial. This can be confirmed by looking on the ORCID statistics web page [https://orcid.org/statistics]. The number of active ORCID users is 7.65 million, whereas only 1.91 million ORCID profiles mention publications. Hence, 75% of profiles do not indicate any publications. These data underline the principal drawback of ORCID. That is, some researchers create a profile on ORCID to access internet services such as submission platforms (e.g. Scholar One) but access them infrequently (e.g. whenever it is required) [22,33] without having to make their profiles public or fill them out. Therefore, the number of publications assigned to ORCID is often lower than expected in these cases [35]. One can legitimately question the utility of having non-public or empty profiles on ORCID because it is in total contradiction with the main objective of this service, which is to allow researchers to reference their publications exhaustively with unambiguous identification of their publications, and to track their scholarly output [13]. The fact that this is all based on what is declared by the researchers themselves greatly limits the interest in using ORCID because it is an unsupervised, self-managed database. Nevertheless, it would be useful to be able to compare our data obtained from a country in which using ORCID is not encouraged in institutions with countries in which its use is encouraged (e.g. Italy or Australia) to see if the number of empty profiles is as high as the highlighted fraction in our study.

Conversely, the average number of publications referenced per researcher available on ResearchGate is generally higher than the number of publications on Scopus (71.1 vs 46.5). Using ResearchGate to assess the number of publications per researcher allows 91.2% of the researchers to maximize this number. On Academia.edu, there is approximately the same number of cases in which the number of publications is higher or lower than on Scopus, with many outliers, causing large discrepancies in some researchers’ statistics.

Apart from ResearchGate, and to a lesser degree ResearcherID, Spearman’s rank correlation coefficient shows a poor correlation between the number of publications and those found on Scopus. Furthermore, due to numerous outliers, results presented in Fig 5 show that using the four sites studied to assess the number of publications per individual is far from being relevant for most researchers because the number of publications depends on the personal motivation of researchers to reference their publications on these sites. It is, however, one of the objectives claimed by these sites. Considering that the presence of researchers on these sites is very limited (except for ResearchGate, see results above), using them to assess output (i.e. the number of publications) per institution or individual, gives results quite different from those obtained using the Scopus database, which is considered to be a reference for calculating scientific output.

### Number of citations per article and h-index

At an institutional level, the total number of citations for articles (number of times the article was cited) found in the profiles of the Caen University researchers was 172,689 on ResearcherID and 513,236 on ResearchGate compared to 657,319 on the Scopus database. We were not able to assess the h-index for institutions for the entire population of researchers because data on overall numbers of citations for each researcher was gathered, and data on the number of citations for each article was not collected.

**Fig 6:**
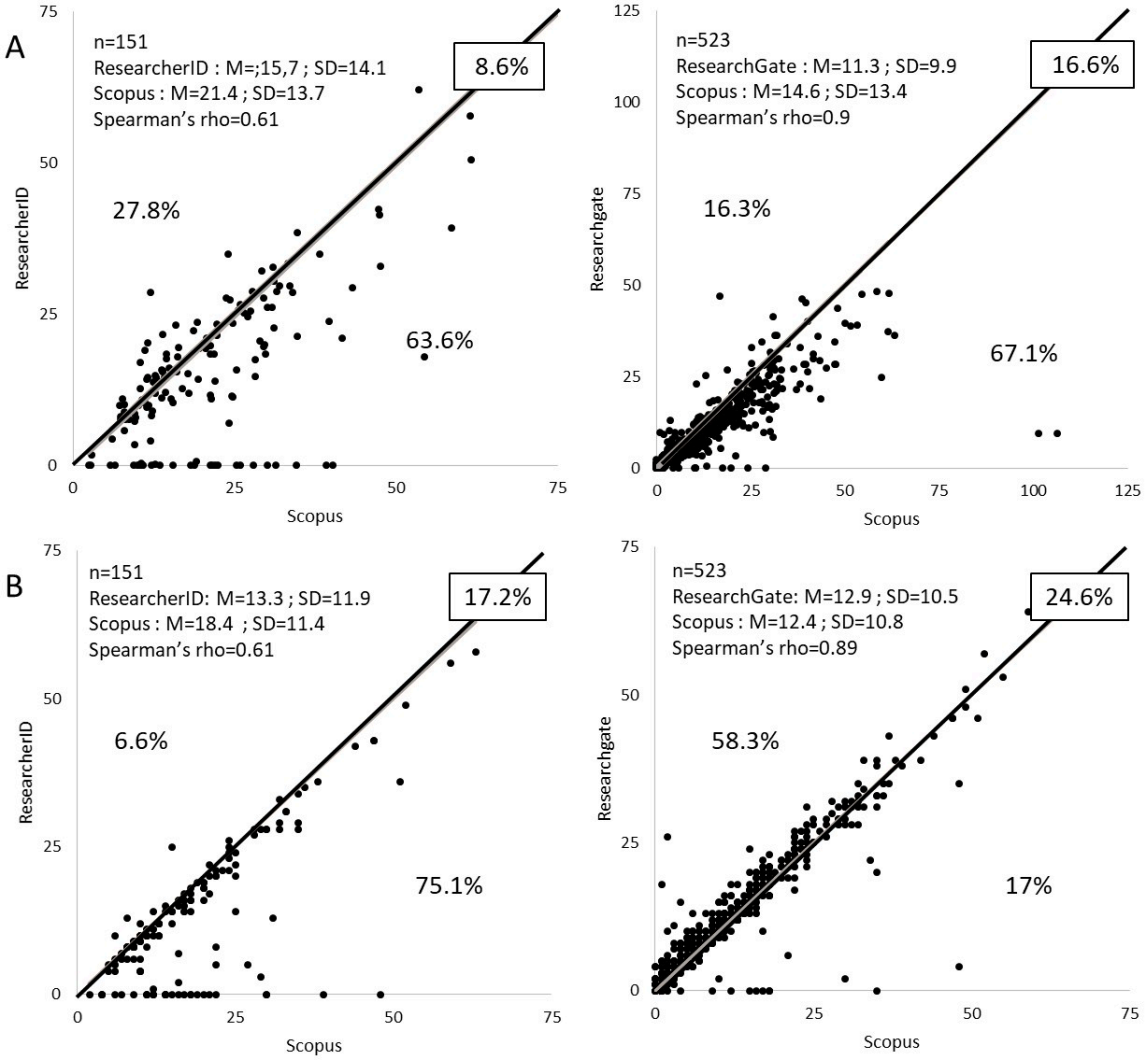
Number of citations per article (A) and h-index (B) for ResearchGate and ResearcherID. Values for citations per article have been rounded to the nearest integer. M and SD: Mean and standard deviation.

For individuals, as shown in Fig 6, Spearman’s rank correlation coefficient shows a strong correlation, especially for ResearchGate, when assessing the number of citations per article and h-indexes compared to Scopus. Nevertheless, considering the number of citations per article on the two sites minimizes this parameter compared to the Scopus database, with many outliers present, particularly for ResearcherID. This corresponds to publications without citations in most cases since they are not referenced in the Web of Science database. Using ResearchGate to assess h-indexes leads to approximately the same average value as for the Scopus database (12.9 vs 12.4). The resulting value for the h-index is identical to that of Scopus for almost a quarter of the researchers (24.6%). Nevertheless, using ResearchGate to assess this parameter for individuals maximizes the h-index for 58.3% of researchers. Conversely, using ResearcherID minimizes this parameter for 75.1% of the researchers.

Our data suggest that assessing the number of citations per article or h-indexes for individuals and institutions using ResearcherID or ResearchGate gives overall results that are quite different from those obtained using the Scopus database. This confirms previous findings which have shown that the usefulness of bibliometric indicators derived from ASNs for institutions [41–43] or individuals [30,37,44] is not clear (results are controversial depending on the ASNs and parameters studied).

## Conclusions

The objective of the present study was to assess the presence of researchers on two AIDs (ORCID and Web of Science ResearcherID) and to compare the results with two ASNs (Academia.edu and ResearchGate) in a medium-sized multidisciplinary university in France (University of Caen Normandy). Few studies have focused on AIDs (Table 4) and, to the best of our knowledge, no one study has been conducted in France on the topic of AIDs and/or ASNs. We believe that a highlight of our study is the manual method used to collect all data analyzed, excluding automatic and informatics methods. This makes the data analysis and results obtained very robust. Our study nevertheless has a major limitation: only one university was included, and although Caen-Normandy University is a medium sized multidisciplinary university, it may not be fully representative of universities in France, and results are clearly not representative of universities found internationally.

Our data show that a large majority of Caen University researchers are not currently interested in using AIDs (75.1%), and to a lesser extent ASNs (40.2%). Moreover, our results show that the two AIDs studied are not presently able to provide the services for which they were created [15] and cannot provide relevant bibliometric metrics for the population studied. Only ResearchGate seems to have the potential to attract researchers, who use it massively and reference their publications on their profiles in significant numbers (on average more than on Scopus), probably at the expense of the other sites. The lack of interoperability between these services prevents researchers from investing in several sites in a relevant manner because it is too time consuming. The large number of profiles without publications found on ORCID seems to be one consequence of this situation. It therefore seems essential for ASNs and AIDs to become more interoperable in the future. However, one can legitimately doubt that a commercial site such as ResearchGate would voluntarily choose to do this at the risk of having its users invest in other sites and leave theirs. Moreover, since the AIDs are so useful to the scientific community, it will certainly be necessary to set up awareness and training events so that researchers will understand the challenges of these tools and why they have to use these sites. If this is not sufficient, it may be necessary to carry out more coercive actions so that researchers invest heavily in these sites, particularly in ORCID, and to take the necessary steps to obtain full and relevant profiles. Only if these conditions are met will AIDs provide the services expected from them. These include addressing the problem of author misidentification, consequently providing exhaustive access to scientific production and bibliometric indicators for individual researchers and their institutions.

## Supporting information

S1 Data (XLSX)
